# Scabies and impetigo in Timor-Leste: A school screening study in two districts

**DOI:** 10.1371/journal.pntd.0006400

**Published:** 2018-05-31

**Authors:** Laura M. Korte, Asha C. Bowen, Anthony D. K. Draper, Kim Davis, Annette Steel, Ines Teodora, Ivonia Mascarenhas, Benjamin Dingle, Joshua R. Francis

**Affiliations:** 1 Paediatric Department, Royal Darwin Hospital, Darwin, Australia; 2 Paediatric Department, Hospital Nacional Guido Valadares, Dili, Timor-Leste; 3 Department of Infectious Diseases, Princess Margaret Hospital for Children, Perth, Australia; 4 Menzies School of Health Research, Charles Darwin University, Darwin, Australia; 5 Wesfarmers Centre for Vaccines and Infectious Diseases, Telethon Kids Institute, Perth, Australia; 6 School of Paediatrics, University of Western Australia, Perth, Australia; 7 Northern Territory Centre for Disease Control, Darwin, Australia; 8 National Centre for Epidemiology and Population Health, Australian National University, Canberra, Australia; 9 Kensington Hill Medical Centre, Leopold, Australia; 10 St John of God Healthcare, Dili, Timor-Leste; Hitit University, Faculty of Medicine, TURKEY

## Abstract

**Introduction:**

Scabies and impetigo are common and important skin conditions which are often neglected in developing countries. Limited data have been published on the prevalence of scabies and impetigo in Timor-Leste. Sequelae including cellulitis, bacteraemia, nephritis, acute rheumatic fever and rheumatic heart disease contribute significantly to the burden of disease.

**Methods:**

School students were recruited from schools in Dili (urban) and Ermera (rural) in Timor-Leste for an epidemiological study in October 2016. A standard questionnaire was used to record demographics, anthropometry and skin examination results. Impetigo and scabies were diagnosed based on clinical examination of exposed surfaces, and clinical photographs were reviewed for correlation by an infectious diseases paediatrician. Prevalence of scabies and impetigo were calculated and binary risk factor associations were described using relative risks and 95% confidence intervals. Adjusted odds ratios were calculated using logistic regression multivariate analysis. Continuous variables were analysed for associations using the Mann-Whitney Rank Sum test.

**Results:**

The study enrolled 1396 students; median age 11 years (interquartile range (IQR) 9–15). The prevalence of scabies was 22.4% (95% CI 20.2–24.7%) and active impetigo 9.7% (95% CI 8.3–11.4%); 68.2% of students had evidence of either active or healed impetigo. Students in Ermera were more likely than those in Dili to have scabies (prevalence 32.0% vs 5.2%, aOR 8.1 (95% CI 5.2–12.4), *p*<0.01). There was no difference in the prevalence of active impetigo between urban and rural sites. More than a third of participants were moderately or severely underweight. Stunting was markedly more common in the rural district of Ermera.

**Conclusion:**

Scabies and impetigo are common in Timor-Leste, with very high prevalence of scabies in the rural district of Ermera. Improvements in prevention and treatment are needed, with prioritised activities in the rural areas where prevalence is highest.

## Author summary

Scabies and impetigo are common and important skin conditions which are often neglected in developing countries. Scabies affects more than 200 million people globally. There are limited data on the prevalence of these conditions in Timor-Leste. Scabies is a parasitic infection which causes an intensely itchy rash in a characteristic distribution. Secondary bacterial infection with group A *Streptococcus* (GAS) and/or *Staphylococcus aureus* causing impetigo is common and may lead to complications. GAS skin infection is known to be associated with the development of acute post streptococcal glomerulonephritis, and may also have a role in the aetiology of acute rheumatic fever (ARF) and rheumatic heart disease (RHD. We assessed almost 1400 school students in Timor-Leste from both urban and rural settings and showed a high prevalence of scabies (22.4%) and active impetigo (9.7%). Two-thirds of students had evidence of either active or healed impetigo. Our study shows that scabies and impetigo are common in Timor-Leste, with very high prevalence of scabies in the rural district of Ermera. Improvements in prevention and treatment are needed, with prioritised activities in the rural areas where prevalence is highest.

## Introduction

Scabies and impetigo are common and important skin conditions which are often neglected in developing countries[1,2]. The global prevalence of scabies was estimated to be over 204 million (in thousands: 204 152 [177 534–237 466])[3], accounting for 0.21% of disability-adjusted life-years from all conditions in the global burden of disease study conducted in 2015[4]. Scabies has recently been adopted as a Category A World Health Organisation (WHO) Neglected Tropical Disease (NTD)[5,6] highlighting the priority of this condition in developing countries. Scabies is a skin infestation caused by the parasite Sarcoptes scabiei. Scabies presents with an intensely pruritic rash with a characteristic distribution pattern[7]. Clinical manifestations include papules, burrows and pruritus [1]. Distribution varies with age and often includes involvement of the webs of the fingers, flexor aspect of the wrist, feet and torso. Transmission is predominantly by direct contact with infected skin, but fomites including bedding and clothing can also play a role [1,8]. Secondary infection by group A Streptococcus (GAS) and/or Staphylococcus aureus causing impetigo is common and may lead to complications including cellulitis, abscess, septic arthritis, osteomyelitis and septicaemia [6]. Impetigo may also occur in the absence of scabies infection due to minor trauma, insect bites and dry skin. GAS skin infection is known to be associated with the development of acute post streptococcal glomerulonephritis, and a role in the aetiology of acute rheumatic fever (ARF) and rheumatic heart disease (RHD) has also been hypothesised [1].

Scabies is endemic in tropical regions globally, with prevalence rates between 5–10% commonly reported in children [9,10]. Timor-Leste is in Southeast Asia and lies northwest of Australia at the eastern end of the Indonesian archipelago. The island is semi-arid with a mountainous terrain and tropical climate. The population of Timor-Leste at the 2015 census was 1.2 million of which 39% were less than 15 years of age. The average household size for Timor-Leste is 5.7 people but is higher in the municipalities of Dili, Alieu, Ermera and Ainaro [11]. Epidemiological data regarding skin infections in Timor-Leste are limited, however previous population screening in Timor-Leste identified scabies in more than a third of children aged under 10 years, as well as high rates of pyoderma coinfection [12].

The aim of this study was to determine the prevalence of scabies and impetigo in school students in urban and rural settings in Timor-Leste and to investigate epidemiological associations of skin disease affecting this cohort. Improved epidemiological evidence is needed in order to design and implement appropriate treatment and prevention strategies at a community level [13].

## Methods

### Ethics statement

Ethics approval for the study was obtained from the Human Research Ethics Committee of the Northern Territory (NT) Department of Health and Menzies School of Health Research (2016–2546) and the Institute Nacional de Saude in Timor-Leste (MS-INS/DF/DP/V/2016/220). Permission to undertake screening was granted by the Ministry of Education in Timor-Leste and by the principals of the schools involved. A plain language information sheet in Tetum (the *lingua franca* in Timor-Leste) was distributed to parents and families prior to commencement of screening, and they were given the opportunity to opt-out of having their child(ren) screened. Use of an opt-out approach to consent occurred because of the strong preference of school principals, clinical staff and other community leaders, identified during informal discussions conducted during consultation visits four months prior to commencement of screening. This is a novel approach to consent in Timor-Leste but has been utilised in similar research in other low and middle-income countries [14,15]. Based on anticipated difficulties with obtaining written consent due to low literacy levels, as well as the low or negligible risk associated with the screening process itself, this approach was approved by both Australian and Timorese human research ethics committees.

Students from schools in the municipalities of Dili (urban) and Ermera (rural) were enrolled in a school screening project that included echocardiography screening for RHD and limited skin examination for scabies and impetigo. The primary outcomes in the skin arm of the study, were presence or absence of scabies, and/or active impetigo, based on limited clinical examination. Baseline demographics including date of birth, age, sex, name, school, address, number of people and number of rooms in the home were collected for all participants. Weight, height and previous known allergy to penicillin were also recorded. Students who attended school on the days of screening were eligible to participate in the study. Children aged less than 5 years and people aged 25 years or older were excluded. Given multiple disruptions to schooling during recent years in Timor-Leste, it is common for secondary students to be aged up to 25 years.

Skin examination was conducted in a school classroom by six medical practitioners who had undergone specific training and validation (using standardised clinical photographs) in the diagnosis of scabies, impetigo and other childhood skin diseases prior to the study. Training was conducted in the week prior to screening. It incorporated teaching about the pathophysiology of childhood skin diseases, descriptions of typical clinical findings, and revision of clinical photographs of varying presentations. Validation involved assessment of 25 clinical photographs of childhood skin diseases, for which candidates were asked to describe their findings and make a diagnosis. Candidates were required to make the correct diagnosis in a minimum of 80% in order to successfully complete training.

Only exposed regions of skin (upper limbs, lower limbs, scalp, face and neck) were examined for reasons of modesty. Students were also asked if they had skin lesions elsewhere on the body hidden under clothing and this was documented but not examined.

Diagnoses of clinical scabies and impetigo were recorded (Box 1). The number of scabies and impetigo lesions were quantified and recorded as ‘none’, 1–10 (mild), 11–49 (moderate), or ≥50 (severe) and body regions involved were documented. [16] The number of inactive (flat, dry) or healed lesions were not quantified. Given the lack of laboratory resources in Timor-Leste it was not feasible to use skin swabs or scrapings for confirmatory diagnosis of aetiological agents.

**Box 1. Diagnostic Criteria for Skin Lesions**

Scabies Presence of burrows, papules, nodules, vesicles, excoriation and/or pruritus in the characteristic distribution.

Crusted Scabies Hyperkeratotic and flaking skin lesions associated with evidence of simple scabies (definition above) in the same patient.

Active Impetigo Papular, pustular or ulcerative lesions with associated erythema, crusting, bullae or frank pus.

Inactive or healed Impetigo Flat, dry impetigo lesions with healed skin, and no signs of active inflammation.

Other skin conditions were also recorded including fungal skin infections, eczema and wounds. Study participants identified as having a skin condition requiring treatment (including scabies and impetigo) were counselled regarding the diagnosis and provided with an information sheet and referral letter to take to the local health clinic for treatment as per standard treatment guidelines.

Anthropometric data were analysed based on WHO normal growth parameters. Weight-for-age (WFA) z-scores were determined for participants aged 5–10 years and height-for-age (HFA) z-score for participants aged 5–20 years. Body-mass-index-for-age (BMIFA) z-scores were calculated for all participants.

Non-identifying clinical photographs were taken of skin lesions to enable later review of clinical diagnoses. Clinical photographs were reviewed by a member of the study team (AB) who is an infectious diseases paediatrician with extensive experience in the diagnosis of scabies and impetigo, who was not present for screening and was blinded to the results of the clinical examination findings obtained during the study.

Data were recorded in an electronic database (Microsoft Access (2016), and analysed using STATA 14.2 (College Station, TX). Prevalence rates of scabies and impetigo were calculated and binary risk factors described using relative risks and 95% confidence intervals (CI). Logistic regression was performed with the following variables in our model: sex, site, age, nutritional status and scabies/impetigo to calculate adjusted odds ratios (aOR) based on multivariate analysis. Continuous variables not normally distributed were analysed for associations using the Mann-Whitney Rank Sum test. Results were considered significant if p<0.05. Subjects with missing data were excluded from analyses of the missing variable.

## Results

A total of 1396 students were enrolled (Table 1). Two-thirds of participants were from the rural area (64.0%) compared to the urban area (36.0%). There were more females (52.8%) than males (47.2%). The median student age was 11 years (interquartile range (IQR) 9–15). The median household size was 7 people (IQR 6–9).

**Table 1:** Demographic characteristics.

	Dili (n = 502)	Ermera (n = 894)	Total (%) (n = 1396)
**Sex**			
Female	254	483	737 (52.8)
Male	248	411	659 (47.2)
**Age (years)**			
5–9	233	179	412 (29.5)
10–14	266	349	615 (44.1)
15–19	3	290	293 (21.0)
20–24	0	76	76 (5.4)
**Anthropometry Median z-score (IQR)**			
Weight for age	–1.48 (IQR –2.24 to –0.83)	–1.79 (IQR –2.46 to –1.14)	–1.65 (IQR –2.36 to –1.01)
Height for age	–0.66 (IQR –1.38 to 0.08)	–1.67 (IQR –2.29 to –1.06)	–1.35 (IQR –2.07 to –0.6)
Body Mass Index for age	–1.78 (IQR –2.67 to –0.89)	–1.19 (IQR –1.86 to –0.56)	–1.39 (IQR –2.15 to –0.67)
**People per household**	Median 6 (IQR 4–8)	Median 8 (IQR 6–9)	Median 7 (IQR 6–9)

IQR: interquartile range, % percentage

Most students were below average for weight, height and body mass index (BMI). Participants from Ermera were more likely to be moderately or severely underweight (WFA z-score <–2) than students from Dili, 40% vs 32%, RR = 1.3 (95% CI 1.0–1.6), p = 0.04. They were also more likely to be moderately or severely stunted (HFA z-score <-2) than participants from Dili, 37% vs 10%, RR = 3.8 (95% CI 2.9–5.1), p<0.01.

Scabies was detected in 312/1396 (22.3%) students screened (Table 2). Of those with scabies, 26.4% had fewer than 10 lesions, 47.9% had 10–49 lesions, and 25.7% had 50 or more lesions. There was one case of crusted scabies identified diagnosed based on clinical findings. Dermatoscopy and skin scrapings were not performed.

**Table 2:** Prevalence of scabies and adjusted odds ratio for variables in multivariate analysis.

	Prevalence (%, 95% CI)	aOR (95% CI), p value—multivariate analysis
**Total**	312/1396 (22.4%, 20.3–24.7)	-
**Sex**		
Female	150/737 (20.4%, 17.6–23.4)	Ref
Male	162/659 (24.6%, 21.5–28.0)	1.3 (1.0–1.8), p = 0.05
**Site**		
Dili	26/502 (5.2%, 3.5–7.5)	Ref
Ermera	286/894 (32.0%, 29.0–35.1)	7.3 (4.6–11.7), p<0.01
**Age (years)**		
5–9	61/412 (14.7%, 11.7–18.5)	Ref
10–14	126/615 (20.5%, 17.5–23.9)	1.1 (0.8–1.7), p = 0.48
15–19	91/293 (31.1%, 26.0–36.6)	1.2(0.8–1.8), p = 0.43
20–24	34/76 (44.7%, 34.1–55.9)	Insufficient data
**Nutrition (n = 1264)**		
Not stunted	161/922 (17.5%, 15.1–20.1)	Ref
Stunted	109/342 (31.9%, 27.2–37.0)	1.3 (1.0–1.8), p = 0.07
**Impetigo**		
No impetigo	245/1260 (19.5%, 17.4–21.8)	Ref
Impetigo	67/136 (49.3%, 41.0–57.6)	4.4 (2.9–6.8), p<0.01

aOR: adjusted odds ratio, CI: confidence interval, % percentage

For the participants documented to have scabies lesions most had more than one body region affected. Scabies lesions were reported on the face/scalp in 14/312 (4.5%) of students, neck 52/312 (16.7%), arms 209/312 (67.0%), hands 271/312 (86.9%), legs 155/312 (49.7%) and feet 145/312 (46.5%), with no significant differences in distribution for the different age groups. Scabies in other regions including torso, buttocks, breasts and groin were self-reported with data recorded for less than half the participants.

Multivariate analysis showed that students in Emera were seven times more likely than those in Dili to have scabies (aOR 7.3 (95% CI 4.6–11.7)). Although more males had scabies, this was not statistically significant (aOR 1.3 (95% CI 1.0–1.8)). The median number of people per house for those with scabies was 8 (range 2–16) versus a median of 7 in the houses without scabies (range 2–13), p<0.01. Students aged 20–24 years old were twice as likely to have scabies than those aged 5–9 years (Table 2). All 20–24 year old students were from Ermera.

Purulent or crusted impetigo was diagnosed in 136/1396 (9.7%) of students screened (Table 3). Of those with impetigo, the vast majority (91.3%) had fewer than 10 lesions.

**Table 3:** Prevalence of impetigo and adjusted odds ratio for variables in multivariate analysis.

	Prevalence (%, 95% CI)	aOR (95% CI), p value—multivariate analysis
**Total**	136/1396 (9.7%, 8.3–11.4)	
**Sex**		
Female	62/737 (8.4%, 6.6–10.6)	Ref
Male	74/659 (11.2%, 9.0–13.9)	1.4 (1.0–2.1), p = 0.07
**Site**		
Dili	41/502 (8.2%, 6.1–10.9)	Ref
Ermera	95/894 (10.6%, 8.8–12.8)	0.9(0.6–1.5), p = 0.77
**Age (years)**		
5–9	36/412 (8.7%, 6.4–11.9)	Ref
10–14	66/615 (10.7%, 8.5–13.4)	1.2(0.7–1.8), p = 0.55
15–19	25/293 (8.5%, 5.8–12.3)	0.7(0.4–1.3), p = 0.31
20–24	9/76 (11.8%, 6.1–21.2)	Insufficient data
**Nutrition (n = 1264)**		
Not stunted	80/922 (8.7%, 7.0–10.7)	Ref
Stunted	40/342 (11.7%, 8.7–15.6)	1.1 (0.7–1.8), 0.60
**Scabies**		
No scabies	69/1084 (6.4%, 5.1–8.0)	Ref
Scabies	67/312 (21.5%, 17.3–26.4)	4.4 (2.9–6.8), p<0.01

aOR: adjusted odds ratio, % percentage, 95% CI: 95% confidence interval

The presence of scabies increased the risk of impetigo infection on univariate and multivariate analyses (RR 2.5 (95% CI 2.1–3.1), p<0.01, aOR 4.4 (95% CI 2.9–6.8), p<0.01). There were no significant differences in risk of scabies for students of different ages or screened at different sites.

**Fig 1:**
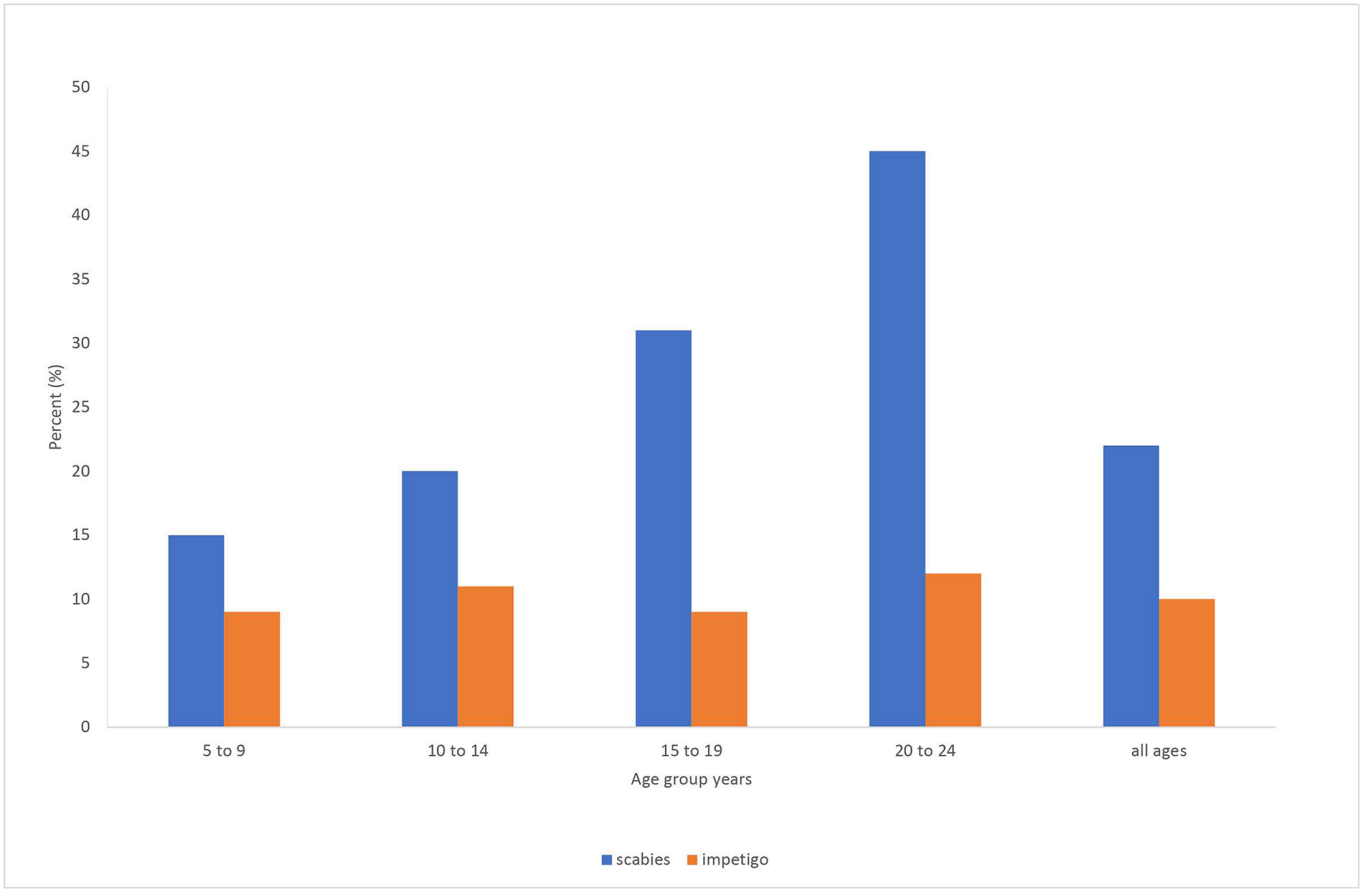
Prevalence of scabies and impetigo by age group.

There was no significant difference in impetigo severity or distribution across the age categories (Fig 1). The distribution of impetigo involving face/scalp and neck was 15/136 (11.0%), arms 29/136 (21.3%), hands 38/136 (28.0%), legs 86/136 (63.2%) and feet 42/136 (30.9%). Most students (68.2%) had evidence of recent impetigo with either active or healed lesions.

Non-identifying clinical photographs of skin lesions were taken from 266 participants (19.1%). Some participants had multiple photos of different types of lesions. Photos of poor quality, without identifying labels, or multiple pictures from the same participant were excluded, with 208 photos available to be reviewed by a paediatric infectious disease specialist with expertise in skin health (AB), who was blinded to the initial screening diagnosis. The sample was representative of the cohort. Concordance of diagnosis was 165/208 (79%) for presence or absence of scabies (Kappa coefficient 0.57 (95% CI 0.46-.067)) and 167/208 (80%) for presence or absence of any impetigo (Kappa 0.55 (95% CI 0.44–0.66)). Agreement was moderate with discordance for impetigo mostly from cases labelled impetigo by the reviewer but not diagnosed clinically, suggesting that clinical examiners may have underdiagnosed impetigo. Disconcordance for scabies was from cases diagnosed clinically but not confirmed by photography, which may relate to difficulties photographing some of the more subtle features of scabies infestation, including burrows.

Other skin lesions were incidentally identified and reported for 168 students. These included warts 59/1396 (4.2%), fungal skin infections 26/1396 (1.9%), injury including lacerations, abrasions, burns and dog bites 24/1396 (1.7%), dermatitis/eczema 19/1396 (1.4%), and cellulitis or abscess 9/1396 (0.6%).

Of the participants identified as having scabies or active impetigo, 95% were referred for treatment at a local clinic. Limited resources were available to treat students with identified severe infection during the screening process. Two students received intramuscular benzathine penicillin G (BPG) for severe impetigo and oral ivermectin was given to 7 students with severe or crusted scabies.

## Discussion

Our estimates of the prevalence of scabies (22.4%, 95% CI 20.2 to 24.7%) and active impetigo (9.7%, 95% CI 8.3 to 11.4%) in Timor-Leste are higher than those reported for school aged children in an earlier study, which found scabies in 159/1114 (14.3%) and pyoderma in 74/1114 (6.6%) [12], and higher than regional norms excluding the Northern Territory of Australia [1,2,9,17,18]. Recent studies in Fiji have also reported very high population prevalence of scabies (36.4%) and impetigo (23.4%).[19]. Recognising this large burden of disease is a necessary first step in developing and implementing strategies to address it.

Scabies is a disease of poverty and associated with household size, low socioeconomic groups and poor access to healthcare have been well documented [1]. Rural students in Ermera were more likely than those in Dili to have scabies. The median number of people per house was 7 in this study. It is likely that scabies infection and household size contribute to ongoing GAS transmission, as suggested by the presence of healed impetigo in the majority of students. Anthropometric data identified that many participants were underweight and/or stunted, particularly in Ermera. These findings are suggestive of poor nutrition and may be indicative of poor socioeconomic conditions.

Scabies and impetigo contribute to a significant burden of disease for children and adults in low resource countries [4,9]. Scabies infection can predispose to secondary bacterial infection with GAS and *S*. *aureus*. GAS is also implicated in the aetiology of glomerulonephritis, ARF and RHD, which can develop in the context of mild infections or carriage. Echocardiography screening, conducted in parallel with the skin screening performed for this study, demonstrated a very high prevalence of definite and borderline RHD (3.5%) amongst school students in these two districts of Timor-Leste [20].

In Timor-Leste, there are limited options for treatment of scabies and impetigo, and consideration should be given to community treatment strategies. First line treatment for scabies is typically with topical agents. Permethrin is effective [21] and well tolerated, however, it is expensive and not widely available in Timor-Leste. In low resource settings sulfur containing preparations and benzyl benzoate 10–25% are often used [1], but regular supply of these is also unreliable. Ivermectin is an oral alternative that has previously been used for refractory cases or crusted scabies and has been trialled successfully for mass drug administration in settings with high prevalence of scabies [13]. Ivermectin has not been used routinely for treatment of scabies in Timor-Leste and is rarely available. Given the high prevalence of scabies in this sample, consideration for mass drug administration is urgently needed. However, the cost, logistics and needed infrastructure make this challenging. Scabies treatment is further complicated by the need to treat household contacts and implement eradication techniques for contaminated clothing and bedding[22].

Oral antibiotics for bacterial skin infection are available at local clinics in Timor-Leste although supply is unreliable. Evidence shows that short course oral trimethoprim/sulfamethoxazole is an effective treatment for impetigo in endemic settings [23], and offers some advantages over BPG injections in terms of tolerability [23,24]. Both antibiotics are cheap, intermittently available and could be used interchangeably in this context based on the high-quality evidence. The impact of this approach on subsequent post streptococcal sequelae such as glomerulonephritis and ARF is unknown.

High rates of skin infection in school students are suggestive of high rates community wide, including vulnerable groups such as infants, preschool children and the elderly. Whilst improving access to clinical treatment is important, consideration should also be given to implementing programs targeted at limiting or eradicating endemic scabies and impetigo. Possible strategies could include community education, water, sanitation and hygiene programs, and community wide treatment with mass drug administration [13].

Strengths of our study include low levels of non-participation. This was likely due to the opt-out consent approach; we were not aware of any families choosing to opt out. Thus, we believe that we have described a representative population of school-attenders in Dili and Ermera districts. The study is obviously limited by not including school aged children and young people who do not attend school which may lead to an underestimate of the prevalence of scabies and impetigo. By limiting the study to these two districts, it is not possible to confidently estimate the prevalence of scabies and impetigo in the rest of the country, but it is likely that rates in other districts are also high. Significantly, more rural than urban students were enrolled in the study. Urban students were enrolled from a single site thus we may not have captured students from all socioeconomic groups in Dili, some of which may have had higher or lower rates of skin infections. Another significant limitation of our study was the limited skin examination as due to the constraints of the facilities and need for privacy only visible skin was examined. This may have resulted in a measurement bias due to the potential under-diagnosis of scabies and impetigo, and the number of lesions on individual students. Clinicians performing skin examination were trained in the identification of scabies and impetigo, but did not receive specific training in the identification of other skin lesions, so these data may not reliably represent the prevalence of other skin diseases in the population. There was no dermatologist on the study team but paediatricians with expertise in the diagnosis and management of skin infections in children were involved.

Scabies and impetigo are highly prevalent in school students in Timor-Leste particularly in the rural district of Ermera. The burden is greater now than it was recognised to be previously. A coordinated approach to improving prevention and treatment are needed, and consideration should be given for implementing strategies at a community level, focusing on rural areas.

## Supporting information

S1 STROBE checklist (PDF)

## References

[1] Hay RJ, Steer AC, Engelman D, Walton S. Scabies in the developing world—its prevalence, complications, and management. Eur Soc Clin Infect Dis. European Society of Clinical Microbiology and Infectious Diseases; 2012;18: 313–323. doi: 10.1111/j.1469-0691.2012.03798.x 22429456 10.1111/j.1469-0691.2012.03798.x

[2] Bowen AC, Mahé A, Hay RJ, Andrews RM, Steer AC, Tong SYC, et al The global epidemiology of impetigo: A systematic review of the population prevalence of impetigo and pyoderma. PLoS One. 2015;10: 1–15. doi: 10.1371/journal.pone.0136789 26317533 10.1371/journal.pone.0136789 PMC4552802

[3] Vos T, Allen C, Arora M, Barber RM, Brown A, Carter A, et al Global, regional, and national incidence, prevalence, and years lived with disability for 310 diseases and injuries, 1990–2015: a systematic analysis for the Global Burden of Disease Study 2015. Lancet. 2016;388: 1545–1602. doi: 10.1016/S0140-6736(16)31678-6 27733282 10.1016/S0140-6736(16)31678-6 PMC5055577

[4] Karimkhani C, Colombara D V., Drucker AM, Norton SA, Hay R, Engelman D, et al The global burden of scabies: a cross-sectional analysis from the Global Burden of Disease Study 2015. Lancet Infect Dis. The Author(s). Published by Elsevier Ltd. This is an Open Access article under the CC BY 4.0 license; 2017;17: 1247–1254. doi: 10.1016/S1473-3099(17)30483-8 28941561 10.1016/S1473-3099(17)30483-8 PMC5700804

[5] Sachs SES, Sachs Ph D JJD, Savioli L, Hotez P, Molyneux D, et al Control of neglected tropical diseases. N Engl J Med. 2007;357: 1018–1027. Available: http://www.nejm.org/doi/full/10.1056/NEJMra064142 17804846 10.1056/NEJMra064142

[6] Engelman D, Kiang K, Chosidow O, McCarthy J, Fuller C, Lammie P, et al Toward the Global Control of Human Scabies: Introducing the International Alliance for the Control of Scabies. PLoS Negl Trop Dis. 2013;7: 5–8. doi: 10.1371/journal.pntd.0002167 23951369 10.1371/journal.pntd.0002167 PMC3738445

[7] Heukelbach J, Feldmeier H. Seminar Scabies. Lancet. 2006;367: 1767–1774. doi: 10.1016/S0140-6736(06)68772-2 16731272 10.1016/S0140-6736(06)68772-2

[8] Mellanby K. The Transmission of Scabies. Br Med J. 1941;2: 406. 20783868 10.1136/bmj.2.4211.405 PMC2162942

[9] Romani L, Steer AC, Whitfeld MJ, Kaldor JM. Prevalence of scabies and impetigo worldwide : a systematic review. Lancet Infect Dis. 2015;15: 960–967. doi: 10.1016/S1473-3099(15)00132-2 26088526 10.1016/S1473-3099(15)00132-2

[10] Steer AC, Jenney AWJ, Kado J, Batzloff MR, La Vincente S, Waqatakirewa L, et al High burden of impetigo and scabies in a tropical country. PLoS Negl Trop Dis. 2009;3: 1–7. doi: 10.1371/journal.pntd.0000467 19547749 10.1371/journal.pntd.0000467 PMC2694270

[11] Government of Timor-Leste. 2015 Population and Housing Census Preliminary Results. 2015; www.statistics.gov.tl/preliminary-results-population-and-housing-census-2015

[12] dos Santos Milena ML, Amaral Salvador, Harmen Sonia P, Joseph Hayley M F JL and C ML. The prevalence of common skin infections in four districts in Timor- Leste: a cross sectional survey. BMC Infect Dis. 2010;10: 61 doi: 10.1186/1471-2334-10-61 20219136 10.1186/1471-2334-10-61 PMC2841184

[13] Romani L, Whitfeld MJ, Koroivueta J, Kama M, Wand H, Tikoduadua L, et al Mass drug administration for scabies control in a population with endemic disease. N Engl J Med. 2015;373: 2305–2313. doi: 10.1056/NEJMoa1500987 26650152 10.1056/NEJMoa1500987

[14] Beaton A, Okello E, Lwabi P, Mondo C, McCarter R, Sable C. Echocardiography screening for rheumatic heart disease in ugandan schoolchildren. Circulation. 2012;125: 3127–3132. doi: 10.1161/CIRCULATIONAHA.112.092312 22626741 10.1161/CIRCULATIONAHA.112.092312

[15] Bhutta ZA. Beyond informed consent. Bull World Health Organ. 2004;82: 771–777. 15643799 PMC2623030

[16] Tikoduadua L, Kama M, Romani L, Steer AC, Tuicakau M, Whitfeld MJ, et al The Epidemiology of Scabies and Impetigo in Relation to Demographic and Residential Characteristics: Baseline Findings from the Skin Health Intervention Fiji Trial. Am J Trop Med Hyg. The American Society of Tropical Medicine and Hygiene; 2017;97: 845–850. doi: 10.4269/ajtmh.16-0753 28722612 10.4269/ajtmh.16-0753 PMC5590570

[17] Mason DS, Marks M, Sokana O, Solomon AW, Mabey DC, Romani L, et al The Prevalence of Scabies and Impetigo in the Solomon Islands: A Population-Based Survey. PLoS Negl Trop Dis. 2016;10: 1–10. doi: 10.1371/journal.pntd.0004803 27348119 10.1371/journal.pntd.0004803 PMC4922659

[18] Andrews R, Kearns T. East Arnhem Regional Healthy Skin Project : Final Report 2008, Cooperative Research Centre for Aboriginal Health, Darwin. 2008; https://www.menzies.edu.au/page/Research/Projects/Skin/East_Arnhem_Healthy_Skin_Project/Publications/5East_Arnhem_Regional_Healthy_Skin_Project_Final_Report_2008/

[19] Romani L, Whitfeld MJ, Koroivueta J, Kama M, Wand H, Tuicakau M, et al The Epidemiology of Scabies and Impetigo in Relation to Demographic and Residential Characteristics: Baseline Findings from the Skin Health Intervention Fiji Trial.: 9–12. 10.4269/ajtmh.16-0753 PMC5590570 28722612

[20] Davis K, Remenyi B, Draper A, Dos Santos J, Bayley N, Paratz E, et al High prevalence of rheumatic heart disease in school students in Timor-Leste: an echocardiography-based prevalence study. Med J Aust. 2017;In Press. 10.5694/mja17.00666 29642817

[21] Taplin D, Porcelain S. Community control of scabies. Lancet. 1991;337: 1548 Available: http://www.ncbi.nlm.nih.gov/pubmed/1675397 10.1016/0140-6736(91)92669-s 1673175

[22] La Vincente S, Kearns T, Connors C, Cameron S, Carapetis J, Andrews R. Community management of endemic scabies in remote aboriginal communities of northern Australia: low treatment uptake and high ongoing acquisition. PLoS Negl Trop Dis. 2009;3: e444 doi: 10.1371/journal.pntd.0000444 19478832 10.1371/journal.pntd.0000444 PMC2680947

[23] Bowen AC, Tong SYC, Andrews RM, O’Meara IM, McDonald MI, Chatfield MD, et al Short-course oral co-trimoxazole versus intramuscular benzathine benzylpenicillin for impetigo in a highly endemic region: An open-label, randomised, controlled, non-inferiority trial. Lancet. 2014;384: 2132–2140. doi: 10.1016/S0140-6736(14)60841-2 25172376 10.1016/S0140-6736(14)60841-2

[24] Andrews RM, Kearns T, Connors C, Parker C, Carville K, Currie BJ, et al A Regional Initiative to Reduce Skin Infections amongst Aboriginal Children Living in Remote Communities of the Northern Territory, Australia. PLoS Negl Trop Dis. 2009;3: 1–9. doi: 10.1371/journal.pntd.0000554 19936297 10.1371/journal.pntd.0000554 PMC2775159

